# A qualitative exploration of variations in social prescribing in primary care for people living with dementia in England: Barriers and enablers to positive service experiences

**DOI:** 10.1371/journal.pone.0361620

**Published:** 2026-10-27

**Authors:** Jenny Corser, Amy Rathbone, Anne Martin, Hannah Barker, Kritika Samsi, Marie Polley, Lucy Clark, Euan Sadler, Emma Wolverson, George Rook, Louise Robinson, Esme Moniz-Cook, Louise Allan, Chris Fox, Jane Cross, Katherine Bradbury

**Affiliations:** 1 Centre for Clinical and Community Applications of Health Psychology, University of Southampton, Southampton, United Kingdom; 2 Faculty of Medicine and Health Sciences, University of East Anglia, Norwich, United Kingdom; 3 School of Health Sciences, University of Southampton, Southampton, United Kingdom; 4 Health and Social Care Workforce Research Unit, Kings College London, London, United Kingdom; 5 Meaningful Measures Ltd, Bristol, United Kingdom; 6 Geller Institute of Ageing and Memory, University of West London, London, United Kingdom; 7 Dementia Action Alliance, Shropshire, United Kingdom; 8 Population Health Sciences Institute, Newcastle University, Newcastle upon Tyne, United Kingdom; 9 Faculty of Sciences, University of Hull, Hull, United Kingdom; 10 Health and Community Sciences, University of Exeter, Exeter, United Kingdom; 11 NIHR Health Technology Research Centre Sustainable Innovation, Exeter, United Kingdom; University of Stirling, UNITED KINGDOM OF GREAT BRITAIN AND NORTHERN IRELAND

## Abstract

The number of people living with dementia and related costs continue to grow worldwide. Although generally associated with memory difficulties, non-cognitive symptoms of dementia are common and usually stem from unmet needs. Evidence emerging suggests that social prescribing improves the wellbeing of people living with dementia. This study explored adaptations to key elements of social prescribing for people living with dementia, as well as barriers to and enablers of positive experiences among people living with dementia and service providers. The qualitative study engaged people living with dementia (n = 8), carers (n = 9) and service providers (n = 17) involved in social prescribing through primary care. Data were collected through semi-structured interviews, and the essential ingredients of social prescribing schemes comprised the initial organising categories for template analysis. The final template included four integrative themes: unique challenges of dementia; interpretations of the social prescribing role; integration of social prescribing into dementia care; and evaluation, improvement and sustainability. Positive workplace cultures and skills in dementia care and support enabled multidisciplinary collaborations and the successful adaptations of the generic social prescribing model to improve the experiences among people living with dementia and service providers. Resistance to and confusion about the social prescribing link workers’ role, and inappropriate referrals were barriers to positive experiences and bottom-up feedback. Primary care networks’ agreements on core outcomes within proactive systems of dementia care and support may promote meaningful bottom-up feedback on the effectiveness of social prescribing for people living with dementia, guiding service improvements.

## Introduction

Dementia is an umbrella term describing a collection of symptoms, including social and cognitive problems, caused by abnormal brain changes [1]. Over 57 million people live with dementia worldwide, with approximately 10 million new cases diagnosed every year [2]. It is estimated that 982,000 people in the United Kingdom (UK) were living with dementia in 2024, costing £42 billion, which is projected to rise to 1.4 million people and £90 billion by 2040 [3]. Although commonly associated with memory difficulties, non-cognitive symptoms of dementia are common and can be behavioural, psychological, or functional challenges, which are often an indication of unmet needs [4]. Emerging evidence suggests social prescribing enhances the wellbeing of people living with dementia [5,6], with a possible social value of £1.17–£5.18 for every pound (£) of investment, demonstrated in peer support for people living with dementia [7].

Social Prescribing is an approach to supporting people living with long-term health needs, such as dementia, by linking them to non-clinical services in the community [8]. In the National Health Service (NHS), social prescribing was introduced as part of the Additional Roles Reimbursement Scheme in 2019 to improve access to services through general practice in England. The funding allocated through Primary Care Networks (PCNs) included limited implementation guidance, which allowed for regional variation in service delivery [9,10]. A PCN brings together a group of general practices working jointly with other health and social care staff to provide proactive and personalised integrated services to a local population [11]. While general practices are not required to be part of a PCN, 99% of general practices in England belong to a PCN due to the significant funding attached [11]. However, PCNs have the autonomy to engage social prescribing link workers themselves or partner with local social prescribing services [10]. Social prescribing link workers work alongside clinicians in general practice to offer 1–6 sessions per person to facilitate a personalised health and wellbeing plan [12,13].

Care planning for people living with dementia in England is integrated in primary care due to the direct access to medication management and referral pathways for supporting physical and cognitive capabilities for as long as possible [14]. Nevertheless, NHS England’s (2020) common outcome framework for social prescribing is not dementia specific, which is compounded by poor consistency and availability of post diagnostic support [15,16]. For example, out of the 276,139 people living with dementia in 85 regions only 41% of people living with dementia were offered social prescribing, of which 8% declined [17].

Qualitative studies exploring social prescribing for people living with dementia identify barriers such as lack of knowledge about social prescribing, service provision limitations, a shortage of specialist knowledge about long term neurological conditions, stigma and anxiety [18–20]. However, little is known about service users’ perspectives of good practice for integrating social prescribing into primary care to improve uptake and enable positive experiences for people living with dementia, their carers and service providers. This study aimed to explore such contextual matters to guide future service improvements, design and implementation by addressing two research questions:

- How are key elements of social prescribing adapted for people living with dementia across England?
- What are the key barriers to and enablers of positive experiences among people living with dementia and service providers?

## Methods

### Study design

The qualitative study conducted in England between October 2024 and February 2025 nestles within a larger, multidisciplinary and cross-regional project (Social Prescribing for People to Live ENjoyably with Dementia/memory problems in Daily Life (SPLENDID) exploring how social prescribing is implemented for people living with dementia across the UK [21]. Normalisation process theory [22] provided a theoretical lens for examining the context in which social prescribing generated particular experiences and their effects on people living with dementia and service providers.

### Ethics approval

Ethical approval was provided by the University of East Anglia (UEA: ETH2324−1932), University of Southampton (ERGO II 98928), and by the Health Research Authority [IRAS ID: 338475]).

### Participants and recruitment

Participants included social prescribing link workers, general practitioners, practice nurses, people living with dementia and their carers. Participants were recruited if they could provide informed consent and if they had experiences of social prescribing through commissioning, making referrals or receiving services within the community through primary care. Recruitment was conducted across England via NHS Research Delivery Networks (RDNs), and electronically allowed snowball participant recruitment via social media, professional networks, and national organisations including:

- National Association of Social Prescribers,
- Social Prescribing Network,
- Together in Dementia Everyday, and
- Join Dementia Research.

Recruitment outreach materials, which contained the inclusion and exclusion criteria invited those interested to contact the research team directly for screening and enrolment in the study. Researchers sent a participant information sheet and consent form via email to eligible participants who expressed interest in taking part in the study. Those wishing to take part returned the forms and researchers booked them in for an interview. All people living with dementia, including those recruited outside of primary care channels were eligible based on self-reports of a formal diagnosis of dementia and having accessed social prescribing services through primary care.

People living with dementia and carers who wished to participate could recall and evaluate past experiences. Exclusions were made for social prescribing services not originating in primary care and referrals related solely to carers rather than people living with dementia. We used the concept of information power [23] to determine the sample size. The inclusion and exclusion criteria ensured that we engaged participants with related experiences and thus greater information power than would be with participants with broad or unrelated experiences [24].

### Data collection

Prior to the interview, each participant provided written informed consent. A brief demographic questionnaire, which was administered verbally and supported by the researcher preceded each interview session. Researchers received verbal consent to record sessions and kept the consent process ongoing for people living with dementia by checking during the interview if they were fine to continue. This enabled researchers to actively monitor any signs of distress or feelings of being pressured to respond to researchers’ questions. All the people living with dementia who participated in interview sessions did not require any form of support before or during the interviews.

A semi-structured interview guide (see supplementary material) with open questions was used to explore social prescribing services participants were involved in. We prompted for service delivery activities, adjustments for people living with dementia by different population characteristics, success stories, challenges, and recommendations for service improvement. Experienced qualitative researchers in health psychology (JCo and AR) conducted one-to-one interviews in person in participant homes (n = 2) and via Microsoft Teams (n = 32). Interview sessions were limited to one hour to respect work and other commitments of participants. Interviews lasted an average of 48 minutes and were recorded, auto transcribed, verified for accuracy and anonymised.

No commissioners were formally interviewed because they did not volunteer to be interviewed individually as participants in the study. Although some of the staff interviewed had some involvement with service commissioning. One commissioner shared perspectives on funding and evaluation during informal recruitment meetings, which were included in the researcher’s field notes to illuminate the interpretation of the data. Participants were offered a £25 gift voucher or had their time reimbursed at the standard rate of the NHS Schedule of Events Cost Attribution Tool [25]. Following interviews, researchers recorded reflections and identified barriers and facilitators to social prescribing service delivery in a shared data log for knowledge sharing and data familiarisation.

### Data analysis

We employed the ‘essential ingredients of social prescribing schemes’ [26], commissioned by NHS England in 2017 as organising codes for template analysis (see Fig 1). Template analysis is a qualitative data analysis technique, which embodies thematic analysis to obtain a balance between structured and flexible coding of data [27]. The essential ingredients of social prescribing schemes [26] originated to provide a foundational model for implementing social prescribing within regional Clinical Commissioning Groups, the bodies with a statutory duty to commission services for local populations. This was selected as the template for the current study because other social prescribing models emphasise social prescribing link worker -patient interaction [28,29]. The community component of the ‘essential ingredients of social prescribing schemes’ was not used to align with the current study’s focus on social prescribing for people living with dementia through their general practitioners. The template is generalised to basic principles of social prescribing broadly rather than describing any form of tailoring for complex conditions such as dementia. This template was a straightforward model to better understand how implementation has been adapted for people living with dementia and how that impacts their experiences of social prescribing.

**Fig 1:**
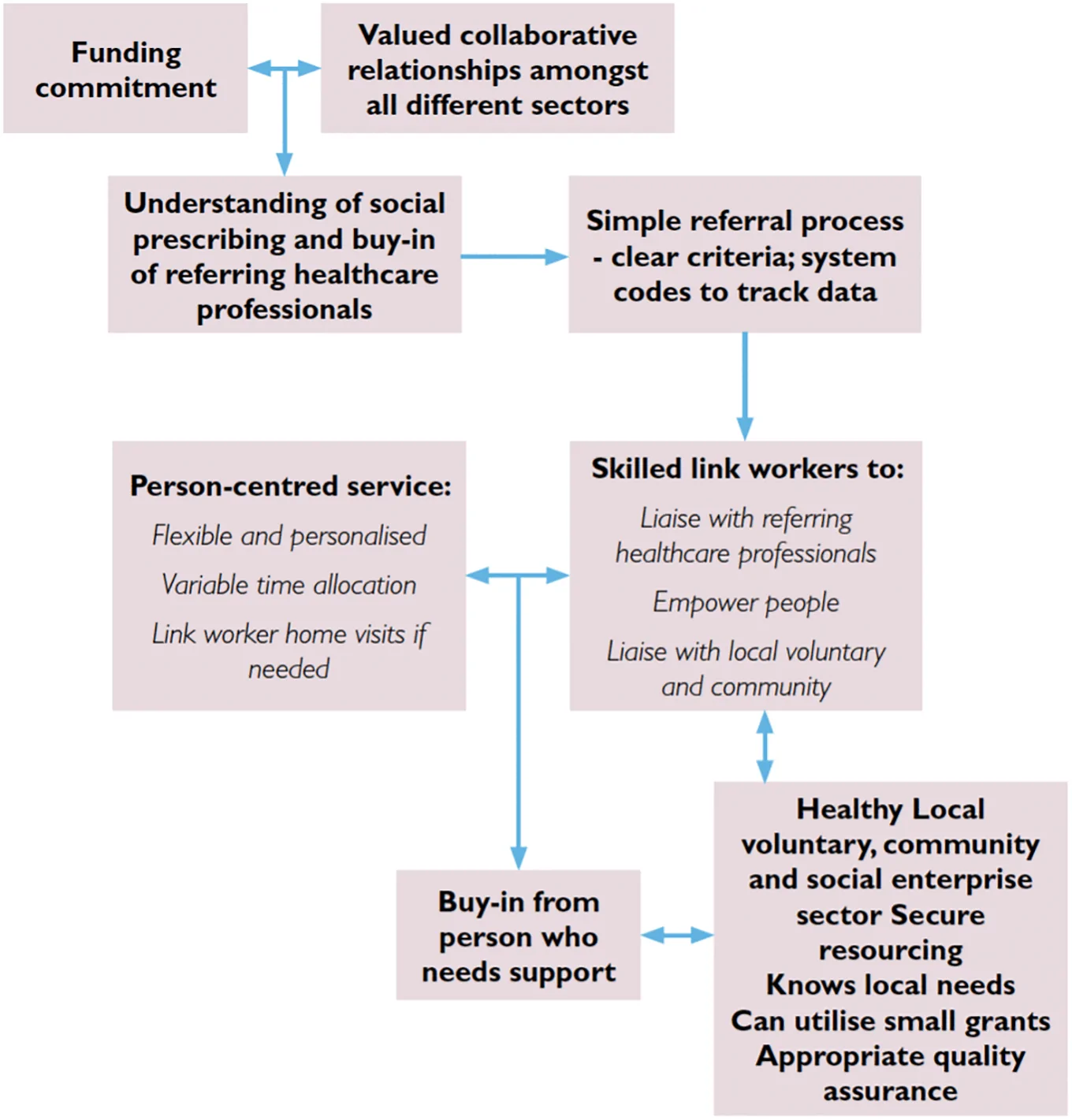
Essential ingredients of social prescribing schemes [26].

One researcher (JCo) used components of Fig 1 as a priori categories to input the data into the deductive template in NVivo 15 and included barriers to social prescribing and good practice categories. Where data did not “fit” new codes and themes were created. The data and ongoing analysis were discussed with experienced qualitative researchers KB, KS, MP, JCr and AR. Normalisation process theory was used as a guiding framework during this stage to sensitise the team to potentially important constructs and to aid the development of integrative themes. These were overarching themes that permeated several categories [23], generated to refine the original template and align with research questions.

Good practice was discussed to distinguish adaptations made in the interest of people living with dementia, which might have also generated positive experiences for service providers. Funding commitment and social prescribing link worker skills were like binding agents shaping all service delivery. Focus could be applied discretely to each ‘ingredient’, although the data largely described interrelationships between categories.

## Results

### Participants

A total of 17 service users were recruited to the study, including people living with dementia (n = 8) and carers (n = 9) (Table 1). The majority (n = 7) of people living with dementia were male, while the majority (n = 7) of carers were female, most (n = 5) of whom were adult children of people living with dementia. Service users identified as either Black British (n = 3) or White British (n = 14), no other ethnicity was recruited. Participant socioeconomic status as measured by postcode showed no participants in the three most deprived deciles of deprivation, despite the relatively fair distribution of participation in terms of rural, coastal, and urban regions. One person living with dementia had lived with the condition for more than ten years, with half of people living with dementia (n = 4) having received the diagnosis in less than a year at the time of the study. A few people living with dementia (n = 3) lived alone with no carer.

**Table 1:** Participant characteristics (service users).

Service Users		People living with dementia n	Carers n
**Total**		8	9
Age	30–39	0	1
	40–49	1	0
	50–59	1	1
	60–69	2	3
	70–79	3	4
	80–89	1	0
Gender	Male	7	2
	Female	1	7
Ethnicity	White British	7	7
	Black British	1	2
	Other	0	0
Time since diagnosis	0–1 years	4	2
	1–10 years	3	5
	10 + years	1	2
Region^a^	Urban	4	3
	Urban/Coastal	1	1
	Rural	1	1
	Coastal	1	1
	Suburban	1	2
Index of multiple deprivation score	1–3	0	0
	4–6	3	3
	7–10	4	4
	unknown	1	2
Care relationship	Person living with dementia no carer/lives alone	3	
	Person living with dementia lives with carer spouse	5	
	Person living with dementia is a parent		5
	Person living with dementia is a spouse		2
	Person living with dementia is a grandparent		2

^a^Self-reported.

A total of 17 service providers were interviewed, including social prescribing link workers (n = 13), General practitioners (n = 2), and Practice nurses (n = 2) (Table 2). All staff identified as White British and worked in a variety of regions. Most (n = 11) staff had been in their current job role for less than 5 years.

**Table 2:** Participant characteristics (service providers).

Staff		n
**Total**		17
Job role	Social prescribing link worker	13
	General practitioner	2
	Practice nurse	2
Gender	Male	5
	Female	12
Ethnicity	White British	17
	Black British	0
	Other	0
Region^a^	Urban	3
	Urban/Coastal	2
	Rural	3
	Coastal	4
	Suburban	5
Length of practice	Less than a year	2
	1-5 years	11
	6-10 years	2
	10 + years	1
	Unknown	1

^a^Self-reported.

Results about adapting key elements of social prescribing for people living with dementia and the barriers to and enablers of positive experiences are organised to correspond to the integrative themes. The integrative theme ‘unique challenges of dementia’ encompasses the needs of people living with dementia and social prescribing link workers and links to all other integrative themes relating to social prescribing adaptations for people living with dementia and their experiences thereof. The final template comprised four integrative themes (Fig 2): (1) unique challenges of dementia; (2) interpretations of the social prescribing role; (3) integrating social prescribing into dementia care; and (4) evaluation, improvement and sustainability. We report enablers of positive experiences as good practice.

**Fig 2:**
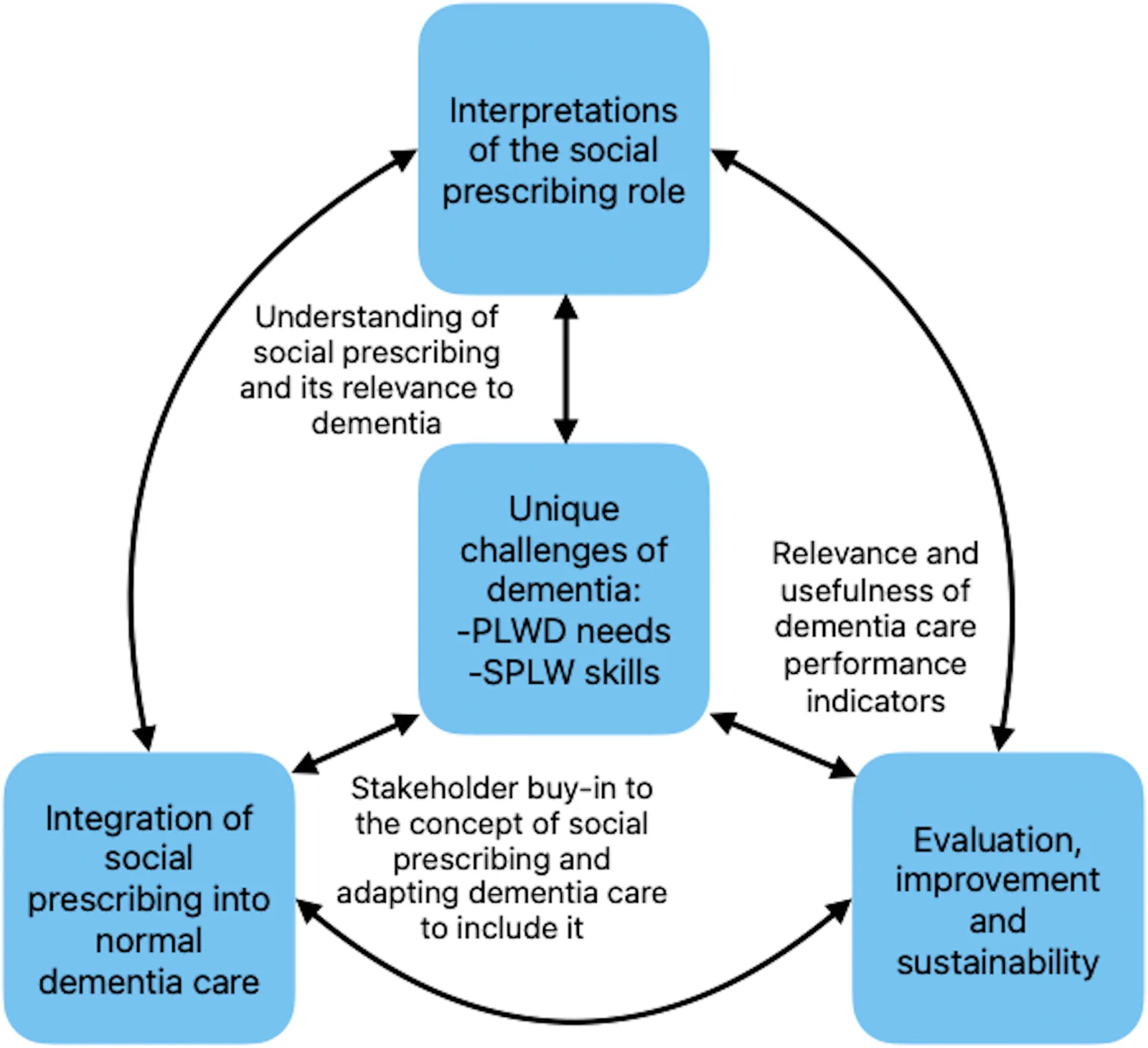
Summary of integrative themes.

### Unique challenges of dementia

#### Person-centred needs.

Dementia care and support present unique challenges that do not fit with the template for social prescribing, especially as far as diagnosis and condition progression are concerned. Consequently, service providers may require additional skills and adaptations to services in general practice. Where general practices were not adapted to the needs of people living with dementia, referrals to social prescribing link workers were often inappropriate as people required dementia specialist support, which reduced social prescribing link workers’ confidence and affected the quality of care. Social prescribing link workers expressing lower confidence tended to focus on the condition and the carers’ needs or simply signposted to lists of local dementia services. Service users and providers commonly reported the signposting approach as impersonal and lacking genuine engagement in supporting the wellbeing of people living with dementia and their readiness to receive information.

“I wanted signposting to services… someone being physically involved in the process…discussing what’s on the leaflet, what is available, what you’re likely to find the experience like.” (Person living with dementia1)

Many service provider participants reported difficulties engaging people living with dementia. These challenges were attributed to people living with dementia experiencing information overload and struggling to grasp the service provider’s role. Examples of people living with dementia not acknowledging the need for or rejecting support were cited. Social prescribing link workers often responded by ending the service and encouraged people living with dementia to call back whenever they needed support. In many cases people living with dementia presented at crisis points due to a lack of awareness of the service, perceptions of not needing support or because of rapidly changing symptoms. Where people living with dementia engaged, other challenges arose in accessing support outside the home, due to social anxieties, accessibility problems (lack of transportation/mobility), or lack of interest in available resources.

“I just, I don’t know really. I just don’t want to go out. I don’t even get dressed most days, I don’t even want to get out of bed, and I just don’t want to walk into a place full of strangers and act like I’m normal…. I should go…. It’s just that first step, I think”. (Person living with dementia7)

#### Good practice.

Good practice focussed on treating people living with dementia as individuals, preserving dignity, and allowing time to connect. This required skill and confidence to see beyond the condition of dementia.

“So, he went in to see her and made to feel like they had all the time in the world”. (Carer 7)

“Let them know you know you’re still Mr. Smith, you know, you’re still a grandfather,”. (Social prescribing link worker 9)

Proactive follow-up by referring staff or social prescribing link workers was identified as important good practice for continued engagement, even after initial resistance.

“People say I’m fine, I’m fine. And then they go home and think for a little while and actually perhaps start thinking because I’m not. So, it’s the opportunity then, for someone to phone them up and say, oh, have you had any more thoughts? can I help in any way?”. (Practice nurse 1)

“If they’re sort of managing OK…I just give the family a call every couple of months just to check that everything’s OK, that nothing’s escalated or”. (Social prescribing link worker 12)

#### Workforce development needs.

Providing authentic person-centred services for people living with dementia required skills such as exploring and recording needs, risk awareness, understanding available supports (when and what), and being sensitive to the emotional impact of a dementia diagnosis and associated vulnerabilities.

“it’s scary, from that moment of diagnosis is scary place because you don’t really know how it’s going to turn or what’s going to happen until it happens. But I guess for us to come in, is that OK? Part of us would be soothing…the process. This is what you need to do. These are people you can go to and hopefully there’ll be groups or support groups or more information”. (Social prescribing link worker 10)

The multi-professional input that is essential in social prescribing necessitated successful collaboration between service providers, as training social prescribing link worker in dementia care and support was not standard practice. Skills required to support people living with dementia were best described by social prescribing link workers with previous personal or professional experience. Where this was lacking, collaboration such as joint home visits with dementia specialists worked well. Through collaboration and direct links with general practitioners and dementia advisors, social prescribing link workers leveraged their skills to optimise the service for people living with dementia.

“But obviously there’s stuff they [dementia advisors] do that I can’t do, you know, and that type of thing. So that’s when [dementia advisor] and I suddenly realised that actually when I need the support for the dementia, let’s do this together, so there’s no repetitiveness, [dementia advisor] will agree. She’ll do this and I’ll do that. And we usually have a little bit of a conversation beforehand. And for us it’s worked amazingly”. (Social prescribing link worker 2)

Specific skills required to support people living with dementia exceeded those in the template for generic social prescribing focused on linking healthcare and community services. Social prescribing for people living with dementia necessitated skill in dementia specific communication and conducting triadic consultations involving carers and people living with dementia. These specialised skills were mostly evident in services with dedicated dementia teams, within a PCN social prescribing link worker group, led by a qualified dementia specialist nurse.

“We have a lot of time to build a rapport … we recognise when someone needs help, and we don’t just then do a referral to somewhere and not see that through. We always make sure that we check in on them again. We also sometimes support people to appointments if we’ve got concerns”. (Social prescribing link worker 11)

### Interpretations of the social prescribing role

#### Lack of clarity of the social prescribing link worker role.

The lack of clarity among referrers and service users about the social prescribing link worker role influenced the ability to adapt social prescribing for people living with dementia. This impacted stakeholder buy-in, collaboration, and the quality of referrals leading to regional service inequalities. Most social prescribing link worker participants understood their role as providing person-centred support for non-clinical needs, identifying individual needs, and linking service users with appropriate services. Their ability to enact this ideal was often constrained by PCN or general practice cultures that expressed limited understandings of the social prescribing link worker role, particularly its application to people living with dementia.

As a relatively new service, most service user participants were unfamiliar with ‘social prescribing’ before referral, which they sometimes encountered under different terms such as community connector. The information they received from referrers, which was often insufficient or misinterpreted primed their expectations.

“I wasn’t entirely sure what we were going for, to be perfectly honest. And because it is social prescriber, I almost thought we’re almost going to get a prescription for so many sessions at some thing or other”. (Carer 7)

Social prescribing link workers discussed needing to advocate for their role among primary care colleagues to improve understanding, referral quality, and their own service delivery. This required them to be confident and tenacious in navigating power dynamics and raising awareness.

“Part of the difficulty is that our roles haven’t been properly discussed with the GPs [general practitioners] and we’re being blocked by admin and operations managers who don’t think it’s relevant… they believe that we just fill out forms. The relationships I’ve built in here is because I’ve knocked on their doors and said “hey. This is who I am. This is what I can do for you. This is how I can support you. Let’s work together”. (Social prescribing link worker 3)

#### The suitability of referrals.

The timing of the referral seemed to influence how people living with dementia experienced social prescribing. Where services were understood to be initiated by people living with dementia or carers as a response to their needs, it frequently became a crisis intervention. Receiving service users in crisis impacted social prescribing link workers’ ability to provide person-centred care, which diminished their confidence in supporting people living with dementia.

“Often what I do see is families getting referred to me when they’re at that crisis point... And at that point then it’s got to the extreme with the person with dementia, so it’s quite difficult for me to deal with them direct at that point. So that’s sort of the stage where I’m more so just dealing with the families”. (Social prescribing link worker 6)

Spontaneous referrals relying on social prescribing as a quick dementia crisis fix or where the social prescribing link worker role was undervalued, resulted in reactive, inconsistent and often inappropriate referrals.

“Most of my referrals, yeah, will be something which is identified during the consultation, which will probably just happen as a natural process. I’m not screening my patients who are coming through to be like, are you a good person to refer to the social prescriber”. (General practitioner 2)

Without proactive screening or pre-emptive referrals, engagement relied on people living with dementia expressing their needs despite their limited awareness of available support, which proved ineffective, particularly where services were poorly defined or advertised. The social prescribing link worker role fell short of a personalised care approach, as people living with dementia and carers did not consider asking for more support or felt it was inappropriate to do so.

“I haven’t tried to do that [ask for support] to be fair… In fact, it never occurred to me if I’m honest, it’s not something I even thought about. I didn’t think you know that they could help”. (Carer 9)

#### Good practice.

Good practice in primary care demonstrated clear referral criteria, including referrals of all people living with dementia based on database codes for diagnosis. Proactive referrals rooted in understanding the social challenges dementia poses, and their impact on quality of life, were identified as good practice. Social prescribing link workers were well placed to establish these supports early. However, even with a clear ethos, inappropriate referrals persisted.

“I commonly, probably once a week at least get a referral because they’ve got memory concerns. Please can you go and assess their cognition. I kindly tell them that’s not part of my role, please send this to the old person’s mental health … I fed it back multiple times. Now I’m going down to the point where I specifically target doctors and tell them this is not our role. Do not send this to us”. (Social prescribing link worker 11)

### Integration of social prescribing into dementia care

#### Mode of introducing the social prescribing link worker role.

Integration of the social prescribing link worker role into general practices impacted the quality of relationships necessary for effective collaboration and social prescribing services, particularly for people living with dementia. Factors suggested to have enhanced collaborative working included individual relationship skills, social prescribing link worker flexibility across multiple sites, and an inclusive workplace culture. Referrer buy-in and interdisciplinary collaboration appeared key to successful integration of the social prescribing link worker role and subsequent incorporation of social prescribing into dementia care. Resistance to the to the top-down introduction of the social prescribing link workers was noted frequently, with some clinicians viewing it as diluting primary care’s ethos and expressing a preference for funding clinical staff according to the medical model.

“I guess it comes down to what you consider to be general practice … If you know that’s done by the Council, the assessment is done by them and I feel like it [funding commitment for SP], you know, should be going through a different route and that shouldn’t necessarily sit with us… I was sceptical of having a social prescriber initially”. (General practitioner 2)

Conversely, people living with dementia buy-in to the social prescribing link worker role appeared to be supported via its association with primary care. Despite the initial social prescribing link worker role ambiguity, service users were open to engaging with social prescribing link workers due to the association with their respected healthcare service provider.

“Social prescribers that work within GP surgeries [general practices] have huge credibility as far as the patients are concerned because the fact that you’re just ringing from the doctor’s surgery, people will open up to you, they’ll talk to you about everything, there’s no barriers there at all and they see that you’re part of the overall care system for them, that’s supporting them… that’s a huge advantage for us”. (Social prescribing link worker 12)

#### Good practice.

General Practices that successfully integrated the social prescribing link worker role hired social prescribing link workers more flexibly and broadened their role beyond simply signposting service users with non-complex social needs to more active integrated team members. Exemplary practice involved adapting the social prescribing link worker role to include dementia reviews. In this example the social prescribing link worker proactively identified people newly diagnosed with dementia, introduced themselves, assessed needs, and shared resources. They also conducted annual dementia review home visits to complete clinical checklists and monitor the wellbeing of people living with dementia.

“When I say I’m a social prescriber, they’d have no clue what that is. So, I have to explain … I tell them that I’m there to signpost to people, to services and community support, anything that they could need… but they also know me as the dementia person … It was never supposed to be part of my social prescribing role…it’s just kind of gone in that direction…because it works, it stayed”. (Social prescribing link worker 5)

### Evaluation, improvement and sustainability

The influence of funding bodies and management system on service delivery, especially through referrer buy-in and referral processes, was clear. However, upstream feedback to stakeholders who funded and set parameters for social prescribing practice was not observed. At general practice level, social prescribing link worker strengths, self-advocacy, patient feedback and multidisciplinary team discussions of the added value influenced referrer buy-in, which appeared naturally over time.

“The longer we’ve had social prescribers, the better it’s got … it did take a little bit of time for, you know, us both to find our feet with each other…them getting more confident in the MDT and more confident in managing patients and us getting more confident in what to give them …it’s worked out pretty well”. (General practitioner 1)

More formal system level evaluation appeared dependent on regional demographic measures where patient referral numbers into social prescribing dictated funding. Proactive (soon after diagnosis) versus reactive (only once a need was identified) referral practices impacted reported numbers leading to funding and staffing complications. For example, reactive referrals tended to be later during the dementia journey, often in crisis situations, which influenced the number of success stories and thus funders’ willingness to continue or expand investment in social prescribing. Where bottom-up feedback back to funders was limited, social prescribing link workers service delivery was hindered, and they reported adapting rules to address people living with dementia needs.

“I bought into the role of social prescribing link worker in its true form and what it was designed to do, and the results are amazing. The PCNs have changed that model… I said I’m not OK with the in-out, in-out, no follow up… I said I need to hang on to some or know the ones that I need to follow because I’ve got to have some success stories”. (Social prescribing link worker 3)

Similarly, performance measures like dementia reviews were sometimes considered checklists for funding rather than quality care interventions. Service users and providers considered performance indicators as arbitrary, causing frustration and negative experiences. The Performance measures intended for service evaluation added burden for staff. While service users perceived these as impersonal.

“But I found that process [of the checklist in the dementia review] soul destroying, so I didn’t go. If that’s all they’re gonna do. I’ve got better things to do with an hour and a half in my afternoon than that”. (Person living with dementia3)

## Discussion

This study explored how the key elements of social prescribing were adapted for people living with dementia across the UK, and the enablers of and barriers to positive experiences among service providers and users. Our results suggest that adaptations depended on social prescribing link workers’ skills in working with and supporting people living with dementia, workplace cultures and the drive to showcase success stories. While inclusive workplace cultures integrated the social prescribing link worker role into existing multidisciplinary teams, clear understanding of the social prescribing link worker role, or lack of, influenced collaborative social prescribing for people living with dementia and the aptness of referrals. Social prescribing link workers’ unfamiliarity with dementia care and support in a non-collaborative environment inadvertently led to impersonal practices and unnecessary disengagement from the service without any meaningful service evaluation. Existing social prescribing performance measures were cited as burdensome and impersonal.

Variations in how the social prescribing link worker role was embedded in primary care showed a postcode lottery, with some providers adapting services to suit the needs of people living with dementia, while others offered standardised social prescribing. These variations are also reflected in regional post-diagnostic dementia care and support, whereby more deprived areas (according to the index of multiple deprivation) have higher health service utilisation rates and higher mortality risks [30]. An evaluation of PCNs found that pre-existing relationships facilitated more straightforward systems for incorporating the Additional Roles Reimbursement Scheme than relationships that had yet to develop trust to work collaboratively [31]. Issues of inappropriate referrals and the need for social prescribing link workers to defend their roles identified here are symptoms of the different operational systems within a PCN, underscoring organisational boundaries. Resisting social prescribing link workers as a watered-down version of general practice distinguishes the fight to cling to the autonomy that general practitioners have long savoured [32]. Where the Social prescribing link worker role was autonomously embraced, its integration into multidisciplinary teams enabled collaborations that prevented ad hoc care and systemic lapses in supporting people living with dementia. Our study adds context to the results of the evaluation of PCNs [31], in which general practitioners’ resistance to employing social prescribing link worker spanned scepticism about the continuity of PCNs and the fluidity of accountability for what and by whom.

People may be blamed for not accepting support when they are expected to manage their own health, yet the real problem is often rooted in wider structural issues [33]. People living with dementia experienced signposting to dementia-specific community activities as a poor attempt at person-centredness, which is essential for social prescribing schemes. Consensus among critiques of the current understanding of person-centredness in dementia care and support [34,35] identifies the need to recognise the evolving selfhood embodied in the reciprocal pleasure of interacting with others. A social prescription for dementia specific group activities may not conform to person-centredness, as that space may engender neither familiarity nor pleasure. Our findings align with the literature [36,37] on the importance of gaining an in-depth understanding of the people living with dementia’s context and working with multiple stakeholders to optimise the benefits of social prescribing. This merits careful thought of the notion that, what is perceived as people living with dementia’s resistance to support may involve a combination of circumstances that social prescribing link workers may need substantial time to unearth.

Contrary to the assumption that social prescribing reduces health inequalities, the service can aggravate inequalities due to factors beyond implicit social determinants of health [37]. Evidence [38,39] suggests that communication barriers in dementia care and support are not limited to people living with dementia but also occur across different care settings, often leading to inequalities in service access. In such cases, the default communication channel is the presenting carer [19], who may not live with the person living with dementia. The pessimism embedded in the concerted focus on the condition of dementia becomes an established barrier to inclusive discussions about choice and control, disempowering what matters to people. Our findings highlighted service users’ expectations of genuine interest in the person and of clear communication that demonstrates respect for people living with dementia, as well as their needs and preferences.

The fixation on people living with dementia’s cognitive decline diverts the responsibility away from providing adequate care and support [33] and refining services to meet users’ needs. While people living with dementia may acknowledge speech limitations, focusing on respectful rather than directive strategies enhance opportunities to establish the rapport necessary for building communication bridges [37]. This is reflected in Reiss et al.’s [40] evidence-based proposition to incorporate adjustments in communication styles to match service users’ needs into standard clinical practice to enhance mutual understanding in dementia care. While the recognition that equipping health workers with dementia training is vital is longstanding [41], dementia training is not mandatory for social prescribing link workers. A qualitative study distinguished social prescribing link workers reactively seek training on dementia to improve their approach to supporting people living with dementia [19]. Social prescribing link worker is a general role, and providing basic training on dementia would be an exemplary adaptation of social prescribing for people living with dementia. As highlighted in our findings, strategies such as making follow-up contact, regular reviews and collaborating with specialists recognise the necessity and proactively put systems in place to support individualised needs.

The social prescribing model [26] presents feedback loops, with hope that a clear understanding of the purpose of social prescribing and the social prescribing link worker role would enable PCNs to define core outcomes and improve transparency. The literature on the broader notion of social prescribing [33,42–44] emphasises that advocacy has outpaced evidence of its impact. Evidence of improved outcomes varies widely, and measures of person-centred care are rarely reported [43]. On the contrary, some provider participants in our study adapted social prescribing to optimise its benefits for people living with dementia in pursuit of successful stories. While the significance of robust evidence on the benefits of social prescribing for people living with dementia is noted [45], crisis intervention referrals may explain the hesitation surrounding regular bottom-up feedback. The current study found that reactive referrals conflicted with the principle of social prescribing as a proactive approach to health and wellbeing and seldom yielded positive outcomes for people living with dementia. A lack of formal evaluation of social prescribing perpetuated a cycle of funding constraints and what social prescribing link workers could offer. Gaining access to the investment and impact fund, which rewards positive performance against targets [31] may depend on the time PCNs need to build effective working relationships.

### Strengths

This study offers a broader lens for understanding existing social prescribing practices for people living with dementia through the perspectives of service users and providers, which may inform interventions to ensure they meet the intended objectives. The findings are embedded in complementary theoretical frameworks, which provide a structured approach to applying concerns and good practice identified on a wider scale. Normalisation process theory [22] provided a useful lens to sensitise the research team to consider a range of factors that shape the delivery of health improvement programmes, facilitating or hindering their integration into usual service delivery.

### Limitations

Participant recruitment was challenging due to service provider workload and service users’ uncertainty about receiving social prescribing through primary care. The method of recruiting service users, which was limited to expressions of interest meeting eligibility criteria rather than purposive demographic sampling, may have constricted the representation of female and non-white ethnicity people living with dementia and spousal carers. Qualitative studies are seldom representative of the wider participant groups and the recruitment approach used in the current study was susceptible to the selection bias of self-selected participant groups. Nevertheless, the study’s inclusion and exclusion criteria may have minimised the selection bias. The digital media which allowed for widespread reach may have excluded people with limited access and/or capacity to use digital technologies. Additionally, the study’s primary care setting excluded views of people who received social prescribing in the voluntary sector.

## Conclusion

Positive workplace cultures, social prescribing link worker role clarity and skill in dementia care and support promoted pre-emptive ways of working that enabled better experiences of social prescribing for people living with dementia than in environments where such elements were lacking. Person-centredness, a key ingredient for social prescribing, ought to shift the focus away from degeneration in dementia support to respect and continuity as people’s experiences of social interactions evolve. This may require flexible social prescribing pathways for people living with dementia to address the health and wellbeing inequalities that present with simply centring cognitive decline. While established internal accountabilities in PCNs are variable [31], agreeing core outcomes within proactive systems of dementia care and support may promote meaningful bottom-up feedback on the effectiveness of social prescribing for people living with dementia, guiding service improvements.

## Supporting information

S1 File Carer interview topic guide. (DOCX)

S2 File Person living with dementia topic guide. (DOCX)

S3 File Practitioner interview topic guide. (DOCX)
