# Supplementary material for: A qualitative exploration of variations in social prescribing in primary care for people living with dementia in England: Barriers and enablers to positive service experiences
Source: PLoS One. 2026 Oct 27;21(10):e0361620. doi: 10.1371/journal.pone.0361620 (PMC13712614; doi:10.1371/journal.pone.0361620)
Supplement: Supplementary file 1 [file pone.0361620.s001.docx]

**SPLENDID Carer Interview Topic Guide**

**A study to evaluate the effect of Social Prescribing (SP) services on lived experiences of People Living with Dementia (PLWD) and their carers**

The following **interview topic guide** will be used with carers of people living with dementia (PLWD) to explore their lived experiences of either their selves, or the PLWD they care for when receiving/participating in social prescribing services, and the subsequent impact that these services may have (if any) on improving their daily living.

The guide includes questions and areas to be explored, with each participant serving as a valuable resource for the research team to draw from during the interviews.

Participant contributions will be fully explored using prompts and probes to understand how and why views and experiences have arisen, hence why no follow-up questions like `why,’ `when,’ `how,’ etc. have been included in this guide.

The sequence in which topics can be addressed is flexible, and the amount of time spent exploring different topics can vary between participants according to individual demographics, clinical factors, and circumstances.

| **Introduction - Key Components**  Thank you  Introduce self  Introduce study  Purpose  Duration  Confidentiality / Anonymity  Limits to confidentiality Opportunity for questions  Consent | Thank you for taking the time to meet with me today.  My name is _________ and I am part of the School of Health Sciences at the University of East Anglia.  We are currently undertaking a research study on social prescribing services for PLWD and carers’ of PLWD.  As part of this study, we are conducting interviews to find out more about your lived experiences of either you, or the PLWD you care for, when receiving/participating in social prescribing services. By exploring your views and experiences, we can get a better understanding of the different social prescribing services currently available for PLWD and their carers’, their value and impact (if any) on improving daily living, and how these services could be improved or supplemented to better address your needs or the needs of the PLWD you care for.  This interview will take up to 90 minutes.  I will be video recording our discussion to make sure that I don’t miss any of your comments - so please be sure to speak up.  Parts of this video recording will be used to produce a video to be used in the main study event (feedback event) to instigate group discussions. This video will be shared with other study participants and the research team only. Outside this context, all responses provided in your interview will be kept confidential and we will make sure that you will not be identified in any way in any of the outputs produced as part of this study.  There are certain limits to confidentiality however: if, during the interviews the research team think that, based on your responses, you, or someone else, is at significant risk of harm, they will have to break confidentiality and discuss with you their intention to pass this information to appropriate third parties.  Please remember that you don’t have to talk about anything you don’t want to and that you can end this interview at any time.  Are there any questions about what I have just explained?  Are you willing to participate in this interview? |
| --- | --- |
| **Questions**  Up to 15 open-ended questions  Use probes when needed:   - Can you please clarify what you meant by …? - Can you please expand a little on …? - Can you please give some examples of …? - What do you think of …? | 1. How much did you know about social prescribing services available for you or the PLWD you care for? How did you learn about them? 2. What specific social prescribing activities or programs have you or the PLWD you care for participated in? What led you to explore this social prescribing activity or program? 3. How has the social prescribing service impacted your quality of life and well-being or the quality of life and well-being of the PLWD you care for? 4. Can you share any specific instances or moments where you observed positive changes or benefits as a result of your or the PLWD you care for participation in social prescribing services? 5. Have you or the PLWD you care for encountered any challenges or barriers when accessing social prescribing services and how were these challenges addressed? 6. Have you or the PLWD you care for encountered any challenges or barriers when utilizing social prescribing services and how were these challenges addressed? 7. How has your role as a caregiver evolved or changed as a result of your involvement or the involvement of the PLWD you care for in social prescribing services? 8. How has your involvement or the involvement of the PLWD you care for in social prescribing services affected your emotional well-being and feelings of as a carer? 9. What aspects of the social prescribing services have been particularly beneficial for you as a carer in terms of support, information, or respite? 10. Can you describe the level of communication and support provided by healthcare professionals or organizations involved in social prescribing and how it has impacted your caregiving responsibilities? 11. How have social prescribing services influenced your overall caregiving experience, including your well-being and stress levels? 12. From your perspective, what are the most valuable aspects of social prescribing services for dementia carers and their loved ones? 13. Can you share any feedback or suggestions for improving social prescribing services to better meet the needs of dementia carers and their loved ones? 14. Have there been any unmet needs or areas where you feel the social prescribing service could be improved to better support PLWD and their carers’? 15. Looking back on your experiences with social prescribing, what recommendations or advice do you have for healthcare providers, policymakers, or organizations involved in delivering these services to dementia carers and their loved ones? |
| **Closing - Key Components**  Additional comments  Next steps  Thank you | Is there anything more you would like to add?  The information collected will be analysed and used in study reports and any other published outputs of the study and part of it will be used to produce the video that will instigate discussions in the main study event (feedback event).  I’ll be happy to send you a copy of our final report if you are interested.  Thank you for finding the time to take part in this interview. |
