# Supplementary material for: A qualitative exploration of variations in social prescribing in primary care for people living with dementia in England: Barriers and enablers to positive service experiences
Source: PLoS One. 2026 Oct 27;21(10):e0361620. doi: 10.1371/journal.pone.0361620 (PMC13712614; doi:10.1371/journal.pone.0361620)
Supplement: Supplementary file 2 [file pone.0361620.s002.docx]

**SPLENDID PLWD (People Living with Dementia) Interview Topic Guide**

**A study to evaluate the effect of Social Prescribing (SP) services on lived experiences of People Living with Dementia (PLWD) and their carers**

The following **interview topic guide** will be used with people living with dementia (PLWD) to explore their lived experiences of receiving/participating in social prescribing services, and the impact that these services may have (if any) on improving their daily living.

The guide includes possible questions and areas to be explored amongst all participants, with each participant telling their own story during the interviews. Dependent on the conversational flow of the interview, each participant may cover 2-3 of the questions suggested.

Participant contributions will be fully explored, using prompts and probes to understand how and why views and experiences have arisen, hence why no follow-up questions like `why,’ `when,’ `how,’ etc. have been included in this guide.

The sequence in which topics can be addressed is flexible, and the amount of time spent exploring different topics can vary between participants according to individual demographics, clinical factors, and circumstances.

| **Introduction - Key Components**  Thank you  Introduce self  Introduce study  Purpose  Duration  Confidentiality / Anonymity  Limits to confidentiality Opportunity for questions  Consent | Thank you for taking the time to meet with me today.  My name is _________ and I am part of the School of Health Sciences at the University of East Anglia.  We are undertaking a research study about social prescribing services for PLWD and carers of PLWD.  As part of this study, we are conducting interviews to find out more about your lived experiences of receiving/participating in social prescribing services. By exploring your views and experiences, we can get a better understanding of the different social prescribing services currently available for PLWD, their value and impact (if any) on improving your daily living, and how these services could be improved or supplemented to better address your needs.  This interview could take up to 90 minutes.  I will be video recording our discussion to make sure that I don’t miss any of your comments - so please try to speak up.  Parts of this video recording will be used to produce a film to be used to instigate group discussions. This video will be shared with other study participants and the research team only. Outside this context, all responses provided in your interview will be kept confidential and we will make sure that you will not be identified in any way as the respondent in any of the outputs produced as part of this study.  There are certain limits to confidentiality however: if, during the interviews the research team think that, based on your responses, you, or someone else, is at significant risk of harm, they will have to break confidentiality and discuss with you their intention to pass this information to appropriate third parties.  Please remember that you don’t have to talk about anything you don’t want to and that you can end this interview at any time.  Are there any questions about what I have just explained?  Are you willing to participate in this interview? |
| --- | --- |
| **Topic areas**  Use probes when needed:   - Can you please clarify what you meant by …? - Can you please expand a little on …? - Can you please give some examples of …? - What do you think of …? | 1. Tell me about any experiences you have had of social prescribing services? How did you learn about them? 2. What specific social prescribing activities or programs have you participated in? 3. What led you to explore this social prescribing activity or program? 4. How has the social prescribing service you participated in impacted your daily life? Please share any positive or negative effects. 5. Could you describe any challenges or barriers you faced when accessing this social prescribing service? 6. Could you describe any challenges or barriers you faced when participating in this social prescribing service? 7. What aspects of the social prescribing service have been particularly beneficial for enhancing your daily living? 8. In what ways has the social prescribing service helped in managing symptoms, maintaining social connections, or improving your quality of life? 9. Have there been any unmet needs or areas where you feel the social prescribing service could be improved to better support PLWD? 10. How would you describe the level of information, and communication provided by the healthcare professionals or organizations involved in social prescribing? What worked well, and what could be improved? 11. How would you describe the level of support provided by the healthcare professionals or organizations involved in social prescribing? What worked well, and what could be improved? 12. Can you share any memorable or significant experiences or moments related to participating in social prescribing services that had a positive impact on your daily living? 13. How has your experience with social prescribing services influenced your perception of dementia care and support within the community? 14. Are there any recommendations or advice you would give to other PLWD considering participating in social prescribing services? 15. Looking back on your experiences with social prescribing, what recommendations or advice do you have for healthcare providers, policymakers, or organizations involved in delivering these services to dementia patients |
| **Closing - Key Components**  Additional comments  Next steps  Thank you | Is there anything more you would like to add?  The information collected will be analysed and used in study reports and any other published outputs of the study and part of it will be used to produce the video that will instigate discussions in the main study event (feedback event).  I’ll be happy to send you a copy of our final report if you are interested.  Thank you for finding the time to take part in this interview. |
