# Supplementary material for: A qualitative exploration of variations in social prescribing in primary care for people living with dementia in England: Barriers and enablers to positive service experiences
Source: PLoS One. 2026 Oct 27;21(10):e0361620. doi: 10.1371/journal.pone.0361620 (PMC13712614; doi:10.1371/journal.pone.0361620)
Supplement: Supplementary file 3 [file pone.0361620.s003.docx]

**SPLENDID Staff Practitioner and Service Commissioning Staff Interview Topic Guide**

**A study to evaluate the effect of Social Prescribing (SP) services on lived experiences of People Living with Dementia (PLWD) and their carers**

The following **interview topic guide** will be used with staff practitioners and service commissioning staff implementing/delivering social prescribing services to people living with dementia (PLWD) and/or carers’ of PLWD.

The guide includes questions and areas to be explored with each participant, serving as a valuable resource for the research team to draw from during the interviews.

Participant contributions will be fully explored using prompts and probes to understand how and why views and experiences have arisen, which is why no follow-up questions like `why,’ `when,’ `how,’ etc. have been included in this guide.

The sequence in which topics can be addressed is flexible, and the amount of time spent exploring different topics can vary between participants according to individual demographics, clinical factors, and circumstances.

| **Introduction - Key Components**  Thank you  Introduce self  Introduce study  Purpose  Duration  Confidentiality / Anonymity  Limits to confidentiality Opportunity for questions  Consent | Thank you for taking the time to meet with me today.  My name is _________ and I am part of the School of Health Sciences at the University of East Anglia.  We are currently undertaking a research study on social prescribing services for PLWD and carers’ of PLWD.  As part of this study, we are conducting interviews to find out more about your lived experiences of implementing/delivering social prescribing services to PLWD and/or carers’ of PLWD. By exploring your views and experiences, we can get a better understanding of the different social prescribing services currently available for PLWD and/or carers’ of PLWD, their value and impact (if any) on improving daily living, and how these services could be improved or supplemented to better address the needs of PLWD and the carers’ of PLWD.  This interview will take up to 90 minutes.  I will be video recording our discussion to make sure that I don’t miss any of your comments - so please be sure to speak up.  Parts of this video recording will be used to produce a video to be used in the main study event (feedback event) to instigate group discussions. This video will be shared with other study participants and the research team only. Outside this context, all responses provided in your interview will be kept confidential and we will make sure that you will not be identified in any way as the respondent in any of the outputs produced as part of this study.  There are certain limits to confidentiality however: if, during the interviews the research team think that, based on your responses, you, or someone else, is at significant risk of harm, they will have to break confidentiality and discuss with you their intention to pass this information to appropriate third parties.  Please remember that you don’t have to talk about anything you don’t want to and that you can end this interview at any time.  Are there any questions about what I have just explained?  Are you willing to participate in this interview? |
| --- | --- |
| **Topic areas**  Use probes when needed:   - Can you please clarify what you meant by …? - Can you please expand a little on …? - Can you please give some examples of …? - What do you think of …? | **Service Implementation:**   1. Can you provide an overview of the social prescribing services you are involved in for PLWD or for carers’ of PLWD? What are the primary objectives of these services? 2. Can you describe the specific activities or interventions offered through social prescribing for this population, and how are they designed to address their needs?   **Service Impact:**   1. Can you share any specific success stories or examples?   **Challenges and Improvements:**   2. What challenges or barriers have you encountered in implementing and delivering social prescribing services for PLWD and/or carers’ of PLWD, and how have you addressed or mitigated these challenges?   **Tailoring Services:**   1. Can you share any specific considerations or adjustments you had to make in service delivery to address specific needs of PLWD and/or carers’ of PLDW? 2. Have you encountered any stigma or resistance to social prescribing within the healthcare system or among potential beneficiaries, and how do you overcome such obstacles? |
| **Closing - Key Components**  Additional comments  Next steps  Thank you | Is there anything more you would like to add?  The information collected will be analysed and used in study reports and any other published outputs of the study and part of it will be used to produce the video that will instigate discussions in the main study event (feedback event).  I’ll be happy to send you a copy of our final report if you are interested.  Thank you for finding the time to take part in this interview. |
